# Weighted Coherence Analysis as a Window into the Neurophysiological Effects of Traumatic Brain Injury

**DOI:** 10.3390/bioengineering11121187

**Published:** 2024-11-25

**Authors:** Ignacio Méndez-Balbuena, Brenda Lesly Betancourt-Navarrete, Ana Cristina Hermosillo-Abundis, Amira Flores, Lucio Fidel Rebolledo-Herrera, Rafael Lemuz-López, Nayeli Huidobro, Roberto Meza-Andrade, Héctor Juan Pelayo-González, María del Rosario Bonilla-Sánchez, Vicente Arturo López-Cortes, Marco Antonio García-Flores

**Affiliations:** 1 Facultad de Psicología, Benemérita Universidad Autónoma de Puebla, Puebla 72000, Mexico; betancourtbl@gmail.com (B.L.B.-N.); ana.hermosilloa@correo.buap.mx (A.C.H.-A.); hector.pelayo@correo.buap.mx (H.J.P.-G.); maria.bonilla@correo.buap.mx (M.d.R.B.-S.); vicente.lopez@correo.buap.mx (V.A.L.-C.); marco.garcia@correo.buap.mx (M.A.G.-F.); 2 Instituto de Fisiología, Benemérita Universidad Autónoma de Puebla, Puebla 72000, Mexico; 3 Facultad de Ciencias Físico Matemáticas, Benemérita Universidad Autónoma de Puebla, Puebla 72000, Mexico; 4 Facultad de Ciencias de la Computación, Benemérita Universidad Autónoma de Puebla, Puebla 72000, Mexico; rafael.lemuz@correo.buap.mx; 5 School of Biological Sciences, UPAEP-CONCYTEP, Puebla 72000, Mexico; nayeli.huidobro@upaep.mx; 6 Departamento de Ciencias de la Salud, Universidad de las Américas Puebla, Puebla 72000, Mexico; roberto.meza@udlap.mx

**Keywords:** TBI, EEG, coherence, Halstead–Reitan categorization task

## Abstract

Traumatic brain injury (TBI), resulting from external forces, is a leading cause of disability and death, often leading to cognitive deficits that affect attention, concentration, speech and language, learning and memory, reasoning, planning, and problem-solving. Given the diverse mechanisms underlying TBI symptoms, it is essential to characterize its neurophysiological and neuropsychological effects. To address this, we employed weighted coherence (WC) analysis in patients performing the Halstead–Reitan categorization task, alongside a control group of eight healthy individuals. Our findings indicate a significant decrease in WC within the theta and delta bands in the temporal regions during cognitive tasks in the TBI group compared to controls. Additionally, we observed a significant increase in WC in the beta and gamma bands in the parietal region during both rest and cognitive tasks in the TBI group, relative to the control group. Furthermore, there was a strong correlation between WC and task performance scores in the temporal regions.

## 1. Introduction

Traumatic brain injury (TBI) is considered the main cause of disability worldwide [1]. Within the first three months following a TBI, patients typically experience impaired brain function. Common issues include altered consciousness, learning difficulties, motor and sensory abnormalities, and intracranial lesions [2,3]. Electroencephalographic (EEG) abnormalities often persist longer than clinical symptoms [4]. In cases of mild TBI (mTBI), attentional complications are the most prevalent, affecting attention span, sustained and selective attention, processing speed, and supervisory attentional control [5,6]. Information processing may slow down permanently after injury [7]. Consequently, attentional function has been proposed as a potential prognostic indicator in TBI patients [8].

Following the acute phase, during the period known as post-concussive syndrome, the management of mild traumatic brain injury (mTBI) often relies on patients’ self-reported accounts, which can introduce subjective bias. Additionally, there is limited evidence that commonly used cognitive tests provide precise and reliable assessments of cognitive dysfunction in these patients [9]. To date, distinguishing between the effects of mTBI and coexisting mental health conditions, whether arising from acute or chronic injury, remains challenging [10,11]. There is currently no objective consensus on how the pathophysiology of chronic TBI relates to its symptoms. Giza and Hovda [12] have proposed mechanisms involving functional and structural changes in neuronal connectivity [13]. However, it remains unclear whether these changes indicate ongoing damage, compensatory reorganization, or the recovery process. This uncertainty poses a significant challenge for the accurate diagnosis and prognosis of TBI [14,15]. Adding to the complexity of prognosis, genetic factors may influence susceptibility to neuropsychiatric disorders frequently comorbid with TBI [16,17]. This highlights the critical importance of personalized medicine in the effective management of TBI.

To address this issue, recent methods for assessing and diagnosing TBI using quantitative EEG (qEEG) have incorporated neural networks [18], machine learning, and measures of spectral power and interhemispheric coherence [19], with varying degrees of success that improve when these techniques are combined. However, it is important to note that, so far, no specific EEG features have been identified that are unique to mild, moderate, or severe TBI. Moreover, current evidence does not support the clinical use of qEEG either immediately after injury occurs or after a significant amount of time, particularly in patients being treated with central nervous system medications.

Given the need for objective diagnostic tools for TBI, a thorough characterization of its electrophysiological effects is essential [20,21]. While anatomical neuroimaging can reveal tissue defects associated with TBI symptoms, such defects are not always detectable in cases of mild TBI. To address this limitation, EEG serves as a non-invasive electrophysiological technique capable of detecting and quantifying sensory and cognitive processing changes at the millisecond level, as observed in TBI [22]. Coherence is a key EEG analysis technique that measures the synchronicity of signals in the frequency domain, enabling the study of functional brain connectivity [23,24,25].

The aim of this study was to assess the neurophysiological and neuropsychological effects of moderate to severe TBI using weighted coherence (WC) in patients performing the Halstead–Reitan categorization task. Given the functional role of neuronal connectivity in cognition, we anticipated that WC would help identify cortical regions with significant connectivity differences between the control and experimental groups, aiding in the identification of diagnostic biomarkers for cognitive disturbances in TBI. We hypothesized that the TBI group would exhibit abnormal WC, particularly in the theta and delta bands, along with hyperconnectivity in high-frequency bands, such as gamma [26].

## 2. Materials and Methods

### 2.1. Participants

The study included eight TBI patients (mean age ± SD, 29.12 ± 8.3 years) and eight healthy controls (mean age ± SD, 29.0 ± 9.01 years). The inclusion criteria for TBI patients were: (1) age between 20 and 50 years with moderate or severe TBI resulting from a traffic accident involving another motor vehicle; (2) at least three months post-injury; and (3) a minimum of ten years of formal education. The control group members were required to be free from any neurological disorders. To account for potential effects on cortical excitability and oscillatory activity, female participants were included at different stages of their menstrual cycle [27]. Lateral dominance was assessed using the Oldfield questionnaire [28]. Socio-demographic variables for both groups are presented in Table 1.

The study was conducted in accordance with the Declaration of Helsinki, and all participants provided informed consent. The research protocol was approved by the Local Ethics Committee of the Faculty of Psychology at BUAP (Protocol Number: NP-001-2015).

### 2.2. Halstead–Reitan Categorization Task

**Figure 1:**
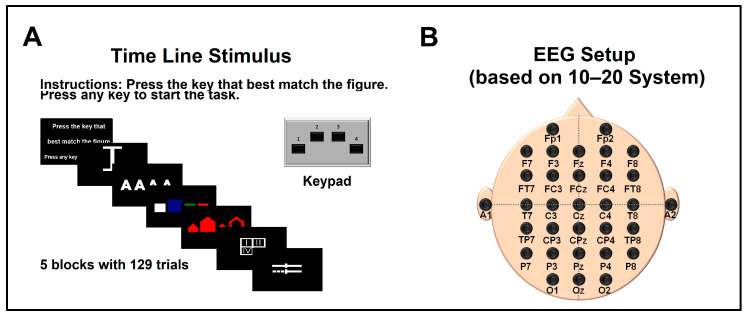
Halstead–Reitan Categorization Task and EEG recording. Panel (**A**) illustrates how the order and timing of stimulus presentation were synchronized with EEG recording to ensure a sufficient window for collecting EEG data during the H-R paradigm. Panel (**B**) shows the electrode placement on the scalp, which followed the international 10–20 system to maintain standardization.

**Figure 2:**
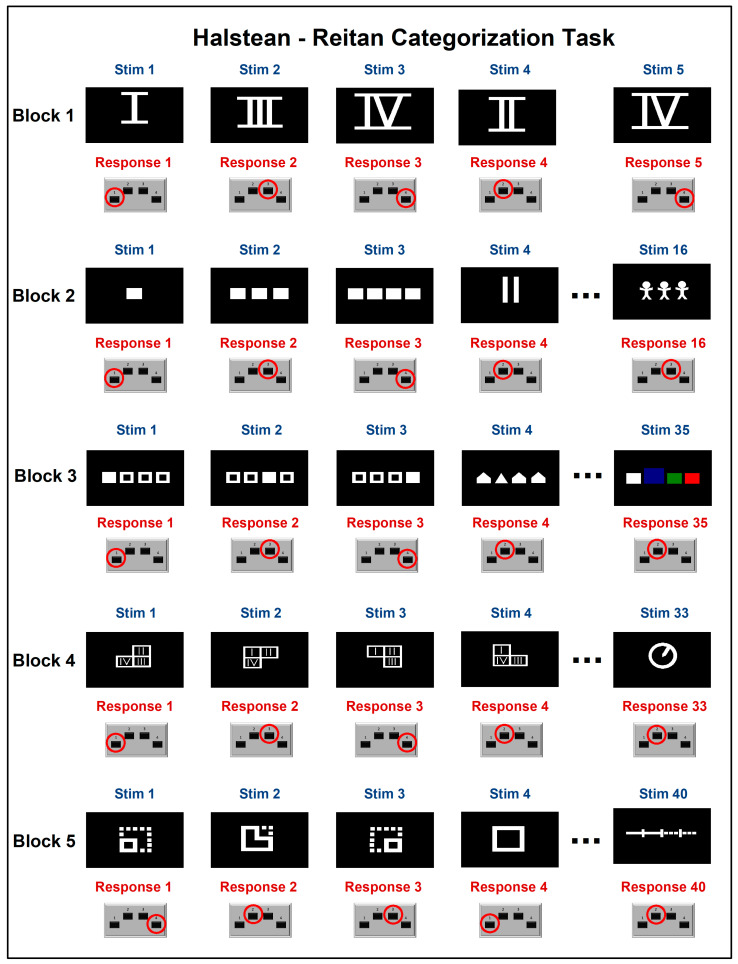
Halstead–Reitan Categorization Task stimuli. The categorization task in NeuroScan Stim consisted of five blocks with a total of 129 images: 5 images in Block 1, 16 in Block 2, 35 in Block 3, 33 in Block 4, and 40 in Block 5. Participants were instructed to press one of four keypad buttons (1, 2, 3, or 4) to select the option that best matched the image stimuli. The figure displays only the first four stimuli and the final stimulus from each block. The correct answer is indicated by a red circle. Note that in Block 1, stimuli 3 and 5 were identical. “Stim” refers to “stimulus”.

Behavioral and electrophysiological data were recorded using NeuroScan v3.0 (Compumedics Neuroscan, Victoria, Australia). The Halstead–Reitan (H-R) categorization task was administered through NeuroScan Stim. The H-R categorization test is a neuropsychological tool designed to assess complex concept formation and abstract reasoning through visual cues [29]. It has been shown to differentiate between varying degrees of TBI severity [30]. The test consists of 167 images divided into seven subtasks, where participants must categorize each image into one of four categories based on experimenter instructions. As the subtasks progress, the images become increasingly abstract and challenging. For this study, we utilized the first five subtasks, which included 129 stimuli (see Figure 1 and Figure 2). Participants received verbal instructions at the start of the experiment, and these instructions were also displayed on the screen at the beginning of each subtask. There was no time limit for responding; participants advanced to the next stimulus by pressing any key on the response pad. They were instructed to press a number (1, 2, 3, or 4) on the response pad that best matched the abstract concept of the image presented. For instance, for an image of the Roman numeral IV, the correct response would be pressing key 4. On average, the test took 15 min to complete. All sixteen participants followed the instructions and reported no fatigue or anxiety during the session.

### 2.3. EEG Data Acquisition

During the experimental session, participants sat comfortably in an electrically shielded, dimly lit room, facing a 24” monitor displaying the stimuli. EEG was recorded with a bandwidth of DC-200 Hz, a sampling rate of 1000 Hz, and electrode impedances maintained below 5 kΩ. A notch filter was applied at 60 Hz (SynAmps, NeuroScan, El Paso, TX, USA). We used a 32-channel Compumedics NeuroScan Quik-Cap to record data from 30 scalp positions, referenced to the earlobes with a ground at FzA, following the 10/20 system. Eye movements and behavioral data were recorded concurrently. The data were stored and analyzed offline.

### 2.4. Data Analysis and Preprocessing

#### 2.4.1. EEG Spectral Power Analysis

During preprocessing, segments contaminated with eye movement artifacts were removed through offline visual inspection. The data were divided into non-overlapping segments, each 1000 ms in duration (with 0 ms marking the start of the task), providing a frequency resolution of 1 Hz for spectral analysis. Preprocessing for EEG spectral power (SP) analysis followed established methods [31,32,33]. The EEG signal was converted to a reference-free current source density (CSD) distribution [34] using the spherical spline interpolation method [35], implemented in Brain Vision 2.0.1 (München, Germany). Each subject’s data included 80 segments of clean EEG. The discrete Fourier transform with 512 points was computed for each segment across the 0–200 Hz spectrum. Data from all Halstead–Reitan paradigm trials and sub-tasks were concatenated for subsequent statistical analysis.

#### 2.4.2. Calculation of EEG Spectral Power and EEG-EEG Cortico-Cortical Coherence

The EEG-EEG cortico-cortical coherence was calculated using methods described in previous studies (Trenado et al., 2014; Méndez-Balbuena et al., 2012; Omlor et al., 2011). Since coherence analysis requires complex values of spectral power (SP), we first computed SP for each channel using the following formula [31,32,33]:

(1) $${SP}_{C}\left(f\right)=\frac{1}{n}\sum_{i=1}^{n}C_{i}(f)C_{i}^{*}(f)$$

Here, C_i_ denotes the Fourier-transformed signal for channel c in a given segment number (n = 1,…, n) and “*” represents the complex conjugate. Coherence values were then computed using the following formula:

(2) $${Coh}_{C1,C2}\left(f\right)=\frac{{\left|S_{C1,C2}(f)\right|}^{2}}{\left|{SP}_{C1}(f)\right|\left|{SP}_{C2}(f)\right|}$$

where

(3) $$S_{C1,C2}\left(f\right)=\frac{1}{n}\sum_{i=1}^{n}C_{1i}\left(f\right){C_{2i}}^{*}\left(f\right)$$

S_C1,C2_(f) is the cross-spectrum for the EEG signal channels C1 and C2 at a given frequency f, while SP_C1_(f), and SP_C2_(f) are the spectral power for C1 and C2 at the same frequency. “*” represents the complex conjugate. Thus, for frequency f, the coherence value, Coh_C1,C2_(f) corresponds to the squared magnitude of a complex correlation coefficient. The function Coh_C1,C2_(f) is a real number between 0 and 1, where 0 indicates absence of synchrony and 1 maximal synchrony between two signals. We considered that coherence was significant if the resulting value lies above the confidence level (CL), defined as follows

(4) $$CL\left(\alpha \right)=1-{\left(1-\alpha \right)}^{\frac{1}{n-1}}$$

where n is the number of segments of 512 points and the symbol α is the desired level of confidence. Coherence was considered significant if it exceeded the 95% confidence threshold. For n = 80 segments and α = 0.95, CL = 0.037.

#### 2.4.3. Regional Weighted Coherence

To quantify EEG coherence, we measured the area under the coherence curve that exceeded the significance threshold. The frequency bands analyzed were Delta (0.5–4 Hz), Theta (4–8 Hz), Alpha (8–13 Hz), Beta (15–30 Hz), and Gamma (30–50 Hz). Coherence values were calculated for all possible pairs of EEG channels within each cortical region, where the number of pairs is given by m(m − 1)/2, with m representing the number of electrodes in the region. The cortical regions analyzed were Frontal (Fp1, Fp2, F7, F3, Fz, F4, F8, FC3, FCz, FC4), Central (C3, CZ, C4), Parietal (P7, P3, Pz, P4, P8), Occipital (O1, Oz, O2), and Temporal (FT7, T7, TP7, FT8, T8, TP8). The sum of all m(m − 1)/2 pairwise coherence values for each electrode in a region R was defined as the Regional Weighted Coherence (RWC_CR_), calculated as follows:

(5) $$RWC=100\times \frac{\sum_{i=1}^{m}\sum_{j=1}^{m}Coh(e_{i},e_{j})}{{Maximun}_{Control\;Group}(\sum_{i=1}^{m}\sum_{j=1}^{m}Coh(e_{i},e_{j}))}$$

where

(6) $${WC}_{j}=\sum_{j=1}^{m}Coh(e_{i},e_{j})$$

is the individual WC for the electrode j, and Coh(e_i_,e_j_) is the coherence between electrodes e_i_, and e_j_.

To visualize the distribution of Regional Weighted Coherence (RWC), we created topographical maps for each cortical region, displaying RWC values for the Delta (0.5–4 Hz), Theta (4–8 Hz), Alpha (8–13 Hz), Beta (15–30 Hz), and Gamma (30–50 Hz) bands.

### 2.5. Statistical Analysis

Statistical analyses were conducted using IBM SPSS Statistics (Version 26, IBM, Armonk, NY, USA). We assessed Local Weighted Coherence during both the H-R categorization task and the eyes-closed resting condition. Comparisons of cortical activity between control and TBI subjects were made across the frontal, central, temporal, parietal, and occipital regions. Due to the lack of homogeneity of variances (Levene’s test, *p* < 0.05) and non-normal distribution of the data (Kolmogorov–Smirnov test, *p* < 0.05), we employed the non-parametric Mann–Whitney U test. This test was used to evaluate whether the RWC differed between control and TBI conditions, with statistical significance set at *p* < 0.05 (two-tailed).

## 3. Results

### 3.1. Regional Weighted Coherence (RWC) Analysis

For each EEG band, we compared Regional Weighted Coherence (RWC) between control and TBI subjects across the five specified cortical regions during the Halstead–Reitan (H-R) task.

**Figure 3:**
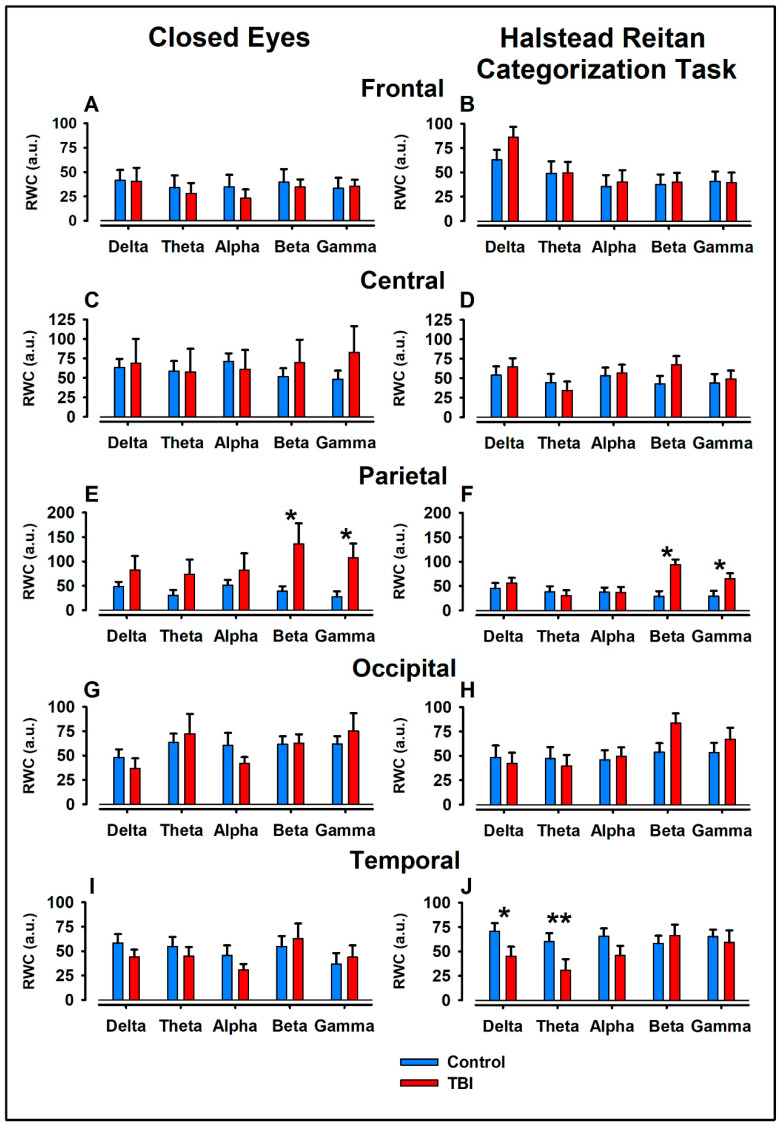
**Regional Weighted Coherence (RWC) for the eyes-closed and Halstead–Reitan Categorization Task conditions.** Panels show RWC values for the frontal (**A**,**B**), central (**C**,**D**), parietal (**E**,**F**), occipital (**G**,**H**), and temporal (**I**,**J**) regions, comparing control (blue) and TBI (red) conditions. RWC values are normalized relative to the control group. Statistical significance is indicated by * (*p* < 0.05) and ** (*p* < 0.01).

In the parietal region, under the eyes-closed condition, we observed significant differences in the beta band: RWC was significantly higher in the TBI group (mean = 136.1) compared to the control group (mean = 38.8), with U = 9, Z = −2.42, *p* = 0.008, r = 0.53. A similar result was found in the gamma band in the parietal region, where the TBI group (mean = 107.3) showed significantly higher RWC compared to the control group (mean = 27.8), with U = 8, Z = −2.52, *p* = 0.006, r = 0.53 (Figure 3E).

### 3.2. Between-Group Contrast

Using the Mann–Whitney U test for between-group comparisons, we found significant differences in RWC values during the H-R task and the eyes-closed condition across the specified cortical regions. In the parietal region, the RWC levels in the beta band were significantly higher in the TBI group (mean = 136.1) compared to the control group (mean = 38.8), U = 9, Z = −2.42, *p* = 0.008, r = 0.53. Similarly in the parietal region, for the gamma band, RWC was significantly higher in the TBI group (mean = 107.3) compared to the control group (mean = 27.8), U = 8, Z = −2.52, *p* = 0.006, r = 0.53 (Figure 3E).

**Figure 4:**
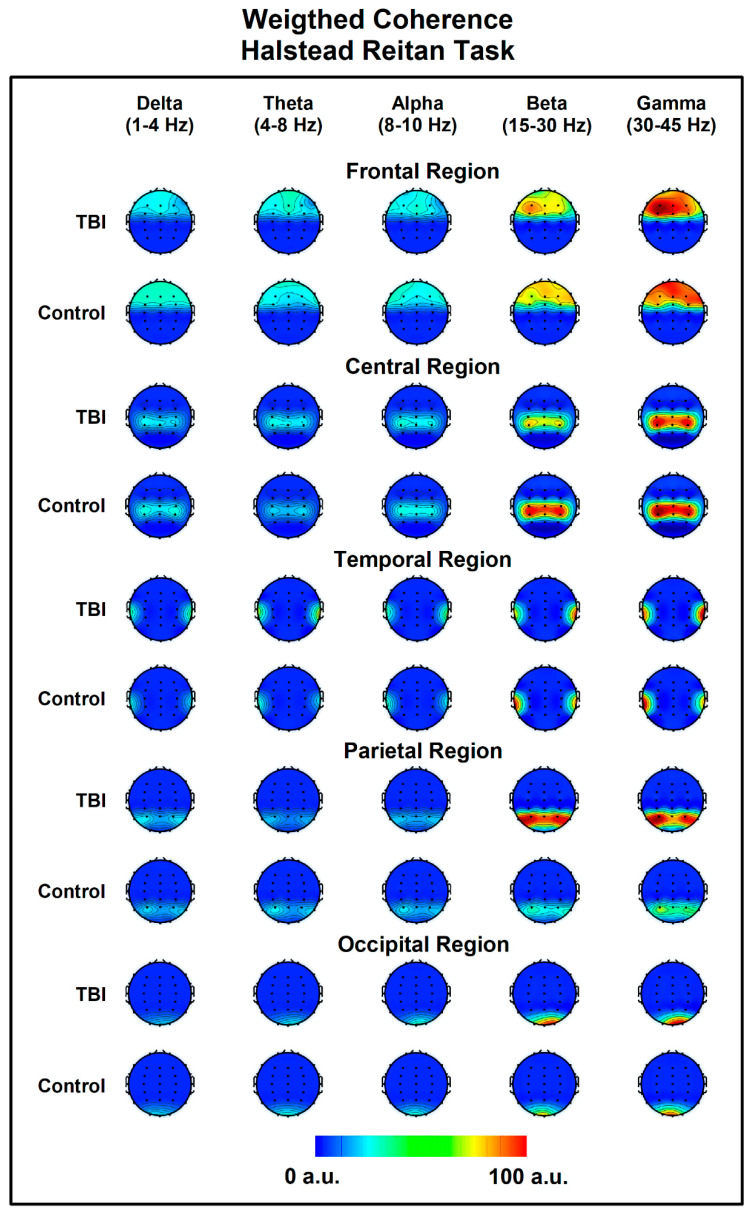
Topographic maps illustrating Regional Weighted Coherence during the Halstead–Reitan Task across different frequency bands: delta (0.5–4 Hz), theta (4–8 Hz), alpha (8–13 Hz), beta (15–30 Hz), and gamma (30–50 Hz). The top row of each panel shows data for the TBI group, while the bottom row represents the control group. Areas of greater coherence are indicated in red, with values approaching 100. The red box highlights the maps for the delta and theta bands in the temporal lobes, where significant differences in weighted coherence between the TBI and control groups were observed.

In the temporal lobes during the H-R task, we found that RWC in the delta band was significantly lower in the TBI group (mean = 44.92) compared to the control group (mean = 70.74), with U = 12, Z = −2.10, *p* = 0.018, r = −0.53. A similar pattern was observed in temporal lobes for the theta band, where the control group (mean = 60.23) had significantly higher RWC compared to the TBI group (mean = 30.68), with U = 12, Z = −2.10, *p* = 0.018, r = −0.53 (Figure 3J). Both quantitatively and qualitatively, these differences in RWC are evident during the HR task, as illustrated in the topographic maps presented in Figure 4.

### 3.3. Intragroup Statistical Tests

For regions and bands showing significant intergroup differences, we conducted Wilcoxon signed-rank tests for intragroup contrasts. In the TBI group, we found a significant difference in the parietal gamma band between the eyes-closed (mean = 107.3) and H-R task (mean = 64.9) conditions, with Z = −1.82, *p* = 0.035, r = 0.45 (Figure 3E,F).

### 3.4. H-R Categorization Task Scores and Latencies

**Figure 5:**
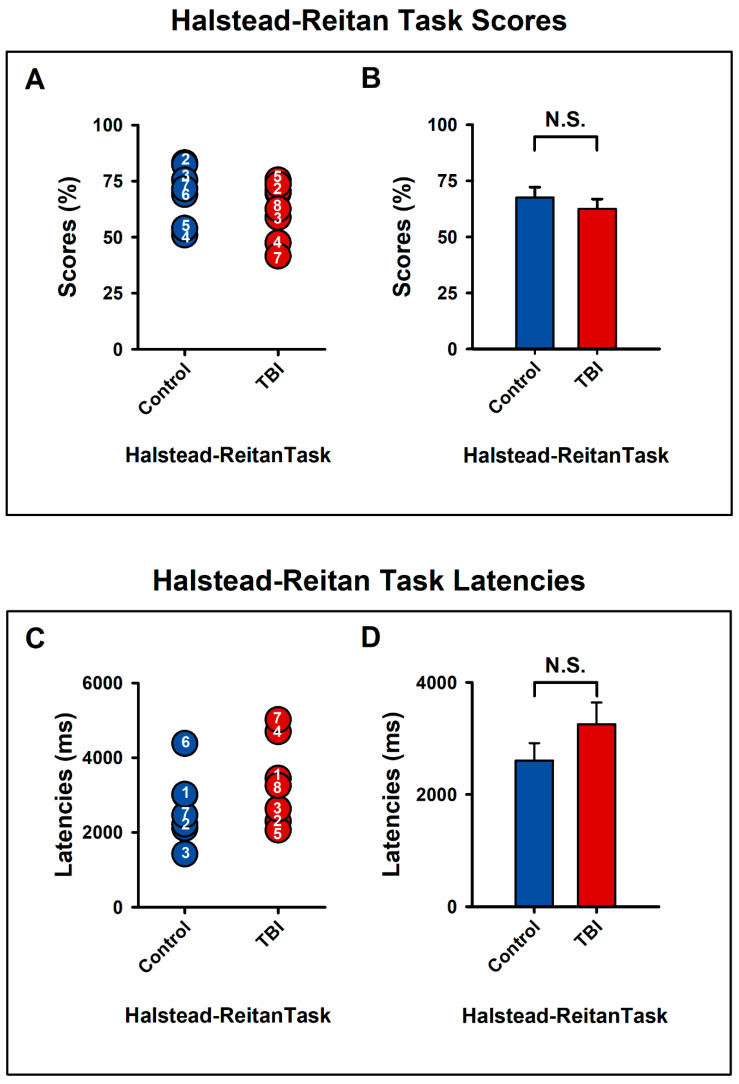
Pooled data showing scores and response latencies for all participants during the Halstead–Reitan categorization task for both the control and experimental groups (**A**,**C**). Control group participants had higher scores, and shorter latencies compared to the experimental group, although these differences were not statistically significant. Panels (**B**,**D**) present the mean values with error bars for each group. N.S. = not significant.

Figure 5A,C show the individual scores (as percentages) and latencies for the Halstead–Reitan categorization task for both the control and TBI groups. The control group scored higher (mean = 70.32%) compared to the TBI group (mean = 66.07%), but this difference was not statistically significant (U = 24.5, z = −0.79, *p* > 0.05, r = −0.2). Similarly, although latencies were shorter in the control group (mean = 2341.2 ms) than in the TBI group (mean = 2942.6 ms), this difference was also not significant (U = 19, z = −1.37, *p* > 0.05, r = −0.34), (Figure 5B,D).

### 3.5. Correlation Between Task Scores and Regional Weighted Coherence

**Figure 6:**
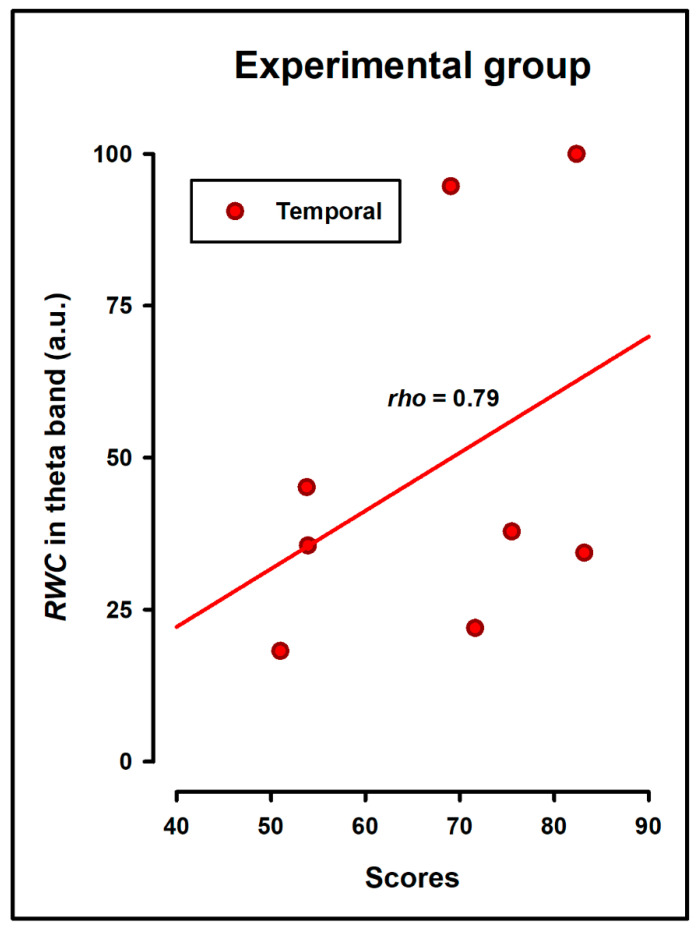
Spearman’s correlation between H-R task scores and Regional Weighted Coherence (RWC) in the experimental group.

In the control group, we observed a significant Spearman’s correlation between task scores and gamma RWC in the occipitoparietal region (r = 0.64, *p* = 0.04). In contrast, the experimental group showed a significant correlation between task scores and theta RWC in the temporal region (r = 0.79, *p* = 0.01), (Figure 6).

### 3.6. General Discussion

This study aimed to identify EEG features specific to traumatic brain injury (TBI) as potential biomarkers for the disorder. We focused on patients with TBI resulting from head-on car collisions, which likely resulted in damage primarily to the frontal or temporal lobes. We assessed Regional Weighted Coherence (RWC) across EEG frequency bands—delta to gamma—within the frontal, central, parietal, occipital, and temporal regions. This approach provided indicators of weighted connectivity and functional brain dynamics, as supported by previous research [23,24,25].

Frontal lobe function is commonly assessed using neuropsychological tests, such as the Halstead–Reitan test and the Wisconsin Card Sorting Test [36,37]. These assessments are applied to various patient populations, including those with dyslexia, ADHD, Parkinson’s disease, and different forms of dementia. Despite its widespread use, the Halstead–Reitan categorization test has faced criticism for its large margin of error and limited specificity in identifying cortical impairments [38]. In this study, we did not use the HR test to assess specific frontal lobe functions. Instead, we selected it because it is designed and standardized to evaluate brain activity primarily associated with the frontal lobe. Thus, we used the HR test as a reliable tool to probe the brain activity of patients with TBI.

Our brain functions as an interconnected network, and damage can alter its structure, function, and connectivity, which may account for the reduced efficiency in brain responses and cognitive processing observed in some TBI patients [39]. Damage to the brain induces significant changes in function and connectivity, which could explain the impaired cognitive processing in TBI patients. To explore these changes, we analyzed brain network communication using weighted coherence (WC), a measure of synchrony between signals from different electrodes within a defined scalp region. A WC of 100% indicates complete synchrony with other electrodes, while a WC of 0% indicates no synchrony. In essence, WC reflects how “connected” or “disconnected” a cortical region is with others. Given the nature of the lesions in our patient sample, we hypothesized that the frontal lobe would show lower coherence compared to other regions. However, our analysis revealed no significant differences in frontal lobe coherence between the experimental and control groups. Instead, we observed higher coherence values in the parietal and occipital regions, particularly in the beta and gamma bands.

Synchronization between brain regions provides insight into the healthy functioning of the brain. Studies have shown that connectivity varies by frequency band: long-distance communication relies on low-frequency bands (delta and theta), while short-distance communication is driven by high-frequency bands (beta and gamma) [39]. Our findings align with this understanding, revealing significant differences between the control and experimental groups in RWC in the low-frequency delta and theta bands within the temporal lobes (left and right), which suggests that there is a decrease in interhemispheric communication in subjects with TBI. This result aligns with the findings of [40], who demonstrated that TBI disrupts interhemispheric functional and structural connectivity.

Interruption of interhemispheric connectivity is strongly associated with mood disorders. Conditions such as anxiety, depression, personality disorders, and post-traumatic stress disorder are notably prevalent among patients with TBI [41]. Neuroinflammatory responses to injury further exacerbate depressive symptoms and anxiety [12]. Additionally, disruptions in serotonergic and dopaminergic systems caused by TBI significantly contribute to these issues [2,15]. Consequently, electrophysiological investigations, such as those conducted in the present study, could serve as valuable clinical indicators for diagnosis, complementing the routine assessment of emotional and psychological factors.

Recent research underscores the value of electrophysiological methods, such as event-related potentials (ERPs) and coherence analysis, in detecting changes in brain function, particularly in patients with subtle cognitive deficits that traditional neuropsychological tests may overlook [11,22,42,43]. One notable change is the loss of synchronization in specific brain areas, characterized by a disruption in rhythmic activity. Desynchronization can result from various factors, including direct damage to components of rhythmic cellular networks, such as subcortical pacemakers, thalamocortical, corticocortical, or cortical networks [44]. Importantly, brain damage in humans is rarely confined to the cortex [45]. In our study, TBI subjects exhibited significant desynchronization in the parietal beta and gamma bands during the categorization task compared to the eyes-closed condition. Despite this, there were no significant differences between the TBI and control groups in these bands and regions. Notably, TBI patients showed significant desynchronization in the temporal lobes in the delta and theta bands compared to controls. This desynchronization may reflect alterations in subcortico-cortical and cortico-cortical functional connectivity following frontal lobe lesions. Our method aims to infer the changes in these circuits caused by TBI.

Maksimenko et al. [46] observed that healthy subjects showed increased cortical beta-band activity in response to ambiguous visual stimuli. High beta-band power is associated with top-down processing and is crucial for disambiguation in the occipital-parietal network and decision-making in the frontal-parietal network [46,47]. The observed higher coherence in TBI patients corresponds with these findings. The control group did not show significant changes in coherence, suggesting that the visual stimuli during the HR test did not challenge normal subjects. In contrast, TBI patients’ slower image interpretation indicates that the temporal and temporo-occipital networks may take on a more dominant role in perceptual decision-making, compensating for the reduced processing speed in the frontal lobe commonly associated with TBI [48,49]. The lack of frontal coherence in TBI patients suggests diminished functional connectivity among frontal neurons, which may account for the cognitive difficulties observed in these individuals, even in cases of mild TBI without visible brain damage. EEG coherence serves as a quantitative measure of dynamic and functional neuronal interactions [50]. Our results suggest that, in heterogeneous TBI populations, frontal neuronal networks may struggle to coordinate effectively, impacting categorization abilities.

In our study, TBI patients demonstrated longer reaction times compared to control subjects, as reflected by the latency between stimulus presentation and response (Figure 5). Although this finding requires validation in future studies with larger sample sizes, it aligns with the established understanding that cognitive fatigue—a hallmark of TBI—is associated with reduced task performance, particularly during activities requiring sustained attention. Cognitive fatigue often results in diminished arousal levels and prolonged reaction times. Consequently, reaction time serves as a reliable measure of processing speed [49,51], which, in this context, can indirectly indicate fatigue due to these underlying factors [52].

We also noted that TBI patients utilized the theta band for communication in the frontal lobe during the HR task. On average, the TBI group used 86.1% of the total communication channels available, compared to 62.8% used by the control group. However, this difference was not statistically significant. Cavanagh and Frank [53] proposed that the frontal theta band serves as a biological marker for neuronal communication, facilitating the separation and transfer of information and organizing neuronal oscillations across different frequencies. Our findings showed that control patients had higher coherence values in the delta and theta bands in the temporal lobe, which may contribute to more stable brain activity organization.

The observed differences in weighted coherence suggest that while the source of brain activity remains similar after TBI, neuronal damage disrupts their functional interactions, impairing distributed processing. This supports the notion that disorganization in brain circuitry underlies the broad cognitive deficits seen in TBI patients, as initially proposed by Luria [54] and further explored in dynamic neural networks [55]. This could also be a mechanism underlying cognitive dysfunction in TBI and particularly in mTBI, as hypothesized by [56].

The Halstead–Reitan test initiates a two-stage perceptual-decision-making process [57,58]. This process involves evaluating sensory data (analyzing visual stimuli), followed by selecting an appropriate response (categorizing images). The engagement of cortical areas varies by time: occipital regions process stimuli within 130–320 ms post-stimulus, while parietal and frontal areas take longer [59]. Perceptual decisions are preceded by activation of a frontoparietal network in the beta-band, which is linked to behavior construction from perception [60]. High-frequency gamma (>50 Hz) and low-frequency beta (15–30 Hz) bands are crucial for network activity, with gamma associated with information encoding and motor planning and beta reflecting local network coordination [58,60]. The frontal theta band, proposed as a “cognitive control mechanism” [53], facilitates transient connections between brain areas, aiding in cognitive organization and information processing. This band has been identified as a predictor of success in executive functioning tasks, influencing action selection, attention shifts, error detection, and behavioral adjustments [53,61,62,63,64]. In executive functioning tasks requiring information maintenance, organization, and inhibition, the alpha band plays a role in suppressing irrelevant stimuli [65]. Our results indicate that the control group exhibited expected synchronizations in the frontal alpha and theta bands, which were significantly higher than those in the TBI group, suggesting that TBI patients have less efficient modulatory mechanisms, requiring more cortical resources and correlating with poorer cognitive control.

### 3.7. Practical Implications

Recent advancements in quantitative EEG (qEEG), including findings from the present study, underscore its growing recognition as a valuable tool for assessing and diagnosing brain damage in TBI patients [66,67]. This represents a significant shift from the uncertainty surrounding its clinical utility just a decade ago [20,43]. By providing sensitive and objective measurements, qEEG can facilitate accurate evaluations and comparisons, supporting the development of more effective, personalized rehabilitation treatments. The ability to measure functional associations among neural populations can help determine the severity of damage and predict cognitive and behavioral changes, enabling tailored rehabilitative strategies.

Traditional neuropsychological tests, while effective for evaluating cognitive and memory functions, often lack the precision needed to assess recovery or progression in TBI patients. A key finding of our study—weighted coherence as a reflection of cognitive processing redistribution—shows promise as a quantitative marker for tracking TBI recovery. When combined with neuropsychological assessments and imaging studies, qEEG emerges as a valuable tool in translational medicine, facilitating the design of personalized treatment strategies [21,22]. Moreover, early therapeutic interventions, such as cognitive rehabilitation and neuroprotective approaches, can significantly enhance neuronal recovery and functional outcomes, as demonstrated by recent advances in neuroplasticity-focused treatments [12,42].

Given its low cost and high informational yield, EEG may also serve as a feasible alternative to costly or inaccessible imaging studies. The straightforward, automatable method for calculating weighted coherence presents an opportunity for creating comprehensive databases on various TBI severities, facilitating the development of interpretable and practical measures to enhance patient care and quality of life.

mTBI impacts different aspects of brain function beyond basic cognitive tasks; for instance, speech or other aspects of language, depending on the cortical areas and/or white matter tracts affected. qEEG allows healthcare professionals to identify the target cortical systems (through weighted coherence, source localization techniques, etc.), as well as expected responses to stimuli (through evoked potentials), beyond the limits of otherwise valuable test batteries. We found that, in patients who received frontal TBIs, the temporo-occipital areas are more active during a cognitive task. An equivalent test using a language-related task (motor, auditory, visual, attentional, etc.) should yield the information that neuropsychologists need to devise the most efficient treatment strategy for each patient.

qEEG has already been used in recent years for the pre-treatment assessment of different language impairments [68], such as aprosodia [69] and aphasia [70,71]. Thus, neuropsychologists could use qEEG findings to develop targeted cognitive rehabilitation programs aimed at improving frontal lobe connectivity, thus optimizing executive functions [22,42].

### 3.8. Limitations and Future Research

This study’s primary limitation is the small, heterogeneous patient sample, which restricts the generalizability of the results. A larger, more homogeneous cohort is needed to validate these findings and identify potential nuances that could influence interpretations. Future research should compare patients across different TBI severities and stages of recovery. Additionally, the absence of baseline EEG recordings prior to the injury limits our ability to measure changes in task performance related to brain connectivity. Future studies could expand the focus to examine connectivity not only at the regional level but also across interregional networks. Such research could explore whether the reduced delta and theta activity in the temporal region in the TBI group is associated with inefficient communication with frontal structures, which are crucial for solving the Halstead–Reitan tasks. Moreover, since the Halstead–Reitan test provides limited information on cortical functions, future studies should use diverse test batteries to corroborate our findings. Selecting the most appropriate test based on dominant cognitive symptoms could enhance diagnostic precision and follow-up care.

## 4. Conclusions

Our findings align with previous research showing differences in quantitative EEG results between control and TBI groups [22], but this study uniquely employs coherence analysis as a methodological approach. The proposed weighted coherence formula revealed significant insights into brain connectivity and damage networks, suggesting that qEEG is a valuable tool for TBI detection and diagnosis, particularly for mild to moderate cases. TBI patients often report changes in their daily functioning despite normal neuropsychological test results. We advocate for incorporating methodologies such as ERPs and coherence measures to better understand brain function and detect alterations in neural communication during complex tasks. Our study demonstrates that brain injury alters neural integration mechanisms, requiring reorganization and increased recruitment of brain areas, leading to diminished synchronization.

## Figures and Tables

**Table 1:** Socio-demographic variables.

Subjects by Group	Sex	Age (Years)	Hand Dominance	Scholarship	TBI Class (Per GCS)	Etiology	Evolution Category	Evolution Time (Months)	Current Drug Use
C1	Female	29	Left	Master	------------	-----------	------------	------------	No
C2	Male	22	Left	High School	------------	-----------	------------	------------	No
C3	Male	24	Right	Bachelor	------------	-----------	------------	------------	No
C4	Male	32	Right	Master	------------	-----------	------------	------------	No
C5	Male	23	Right	Bachelor student	------------	-----------	------------	------------	No
C6	Male	20	Right	Bachelor student	------------	-----------	------------	------------	No
C7	Female	32	Right	Bachelor	------------	-----------	------------	------------	No
C8	Male	50	Right	Bachelor	------------	-----------	------------	------------	No
E1	Female	32	Left	Bachelor	Severe	Motor Vehicle	Chronic	16	No
E2	Male	20	Left	Bachelor student	Severe	Motor Vehicle	Chronic	7	No
E3	Male	24	Right	Bachelor	Moderate	Motor Vehicle	Acute	3	No
E4	Male	30	Right	Bachelor	Moderate	Motor Vehicle	Chronic	13	No
E5	Male	25	Right	Bachelor	Moderate	Motor Vehicle	Chronic	21	No
E6	Male	22	Right	Bachelor student	Moderate	Motor Vehicle	Chronic	25	No
E7	Female	32	Right	Bachelor	Severe	Motor Vehicle	Acute	3	No
E8	Male	48	Right	Bachelor	Moderate	Motor Vehicle	Chronic	28	No

## Data Availability

The raw data supporting the conclusions of this article will be made available by the authors without undue reservation.
